# Evaluation of an Extended Autocorrelation Phase Estimator for Ultrasonic Velocity Profiles Using Nondestructive Testing Systems

**DOI:** 10.3390/s16081250

**Published:** 2016-08-09

**Authors:** César Yutaka Ofuchi, Fabio Rizental Coutinho, Flávio Neves, Lucia Valéria Ramos de Arruda, Rigoberto Eleazar Melgarejo Morales

**Affiliations:** 1 Graduate School of Electrical Engineering and Computer Science (CPGEI), Federal University of Technology-Paraná (UTFPR), Avenida 7 de Setembro 3165, Curitiba 80230-901, Paraná, Brazil; fabiocoutinho@utfpr.edu.br (F.R.C.); neves@utfpr.edu.br (F.N.J.); lvrarruda@utfpr.edu.br (L.V.R.A.); 2 Mechanical & Materials Engineering Postgraduate Program (PPGEM), Federal University of Technology-Paraná (UTFPR), Avenida 7 de Setembro 3165, Curitiba 80230-901, Paraná, Brazil; rmorales@utfpr.edu.br

**Keywords:** ultrasonic velocity profile, autocorrelation, cross-correlation, rotating cylinder, fluid dynamics, dealising signal processing

## Abstract

In this paper the extended autocorrelation velocity estimator is evaluated and compared using a nondestructive ultrasonic device. For this purpose, three velocity estimators are evaluated and compared. The autocorrelation method (ACM) is the most used and well established in current ultrasonic velocity profiler technology, however, the technique suffers with phase aliasing (also known as the Nyquist limit) at higher velocities. The cross-correlation method (CCM) is also well known and does not suffer with phase aliasing as it relies on time shift measurements between emissions. The problem of this method is the large computational burden due to several required mathematical operations. Recently, an extended autocorrelation method (EAM) which combines both ACM and CCM was developed. The technique is not well known within the fluid engineering community, but it can measure velocities beyond the Nyquist limit without the ACM phase aliasing issues and with a lower computational cost than CCM. In this work, all three velocity estimation methods are used to measure a uniform flow of the liquid inside a controlled rotating cylinder. The root-mean-square deviation variation coefficient (*CV_RMSD_*) of the velocity estimate and the reference cylinder velocity was used to evaluate the three different methods. Results show that EAM correctly measures velocities below the Nyquist limit with less than 2% *CV_RMSD_*. Velocities beyond the Nyquist limit are only measured well by EAM and CCM, with the advantage of the former of being computationally 15 times faster. Furthermore, the maximum value of measurable velocity is also investigated considering the number of times the velocity surpasses the Nyquist limit. The combination of number of pulses and number of samples, which highly affects the results, are also studied in this work. Velocities up to six times the Nyquist limit could be measurable with CCM and EAM using a set of parameters as suggested in this work. The results validate the use of the NDT tool to measure velocities even beyond Nyquist limit by using EAM.

## 1. Introduction

Interest in knowing the instantaneous velocity profile in fluid dynamics has grown in recent years as new flow visualization techniques are improving. Optical techniques such as PIV/PTV can measure a large number of velocity vectors simultaneously, however, they are limited to transparent liquids and have cost and high computational load issues. Another established method to measure velocities is the ultrasonic velocity profiler, also called UVP or ultrasonic Doppler velocimetry UDV. This technique has desirable characteristics as it being non-invasive, working with opaque liquids, and the equipment is also portable and easy to install if compared with other velocity profiler methods. The first applications of such method were in blood flow measurement for medical diagnosis. After the 1990s, the technique was also applied in fluid flow studies [1,2] such as magnetic fluids [3,4], hydraulic research [5], multiphase flow [6,7], liquid food and a variety of chemical solutions [8,9].

The idea of UVP is to receive the reflected echoes from tracers inside the flow and use the time or phase delay (Doppler shift) during each ultrasonic emission to estimate flow properties or behaviors. A one-dimensional velocity profile is obtained along the transducer measurement line as a function of time. A basic system for UVP has an ultrasonic transducer excited by a high voltage pulser that also receives the ultrasonic wave. The analog signal is digitized and processed by a signal-processing unit that may be an embedded system or a PC software. The velocity is usually estimated using the autocorrelation method (ACM) that measures the phase delay between ultrasonic emissions using narrowband pulses. The advantage of using this technique is to assure a better signal-to-noise-ratio (SNR) and a low variance by increasing the pulse duration and thus the transmitted energy [10]. One disadvantage of such method is a poorer axial resolution due to the longer pulse duration. Recently, pulse compression signal processing techniques which uses broadband pulses have been used for velocity estimation [11,12]. In this method, linear chirps (frequency modulation) or phase coding pulses are transmitted, keeping the same energy ratio as a narrowband pulse. After a proper filtering, the received signal is still broadband, improving the axial resolution. Both techniques require hardware pulse generation with at least controlled pulse width and number of cycles (narrowband pulser), chirp or coded signal generation (broadband pulser). Those systems are usually designed only for specific applications such as flow measurement systems or medical ultrasonic systems. Only few manufacturers produce those devices which are also so expensive that they may be not affordable for many researchers or industries. An alternative to those pulsers is the use of ultrasonic nondestructive testing (NDT) system pulsers.

NDT systems are widely available since the 1950s [13], and they are used for numerous testing purposes. An existing NDT system could be shared with NDT testers, and enable flow researchers to have a first contact with the ultrasonic velocity profiler technique. However, such systems are designed to generate shorter pulses (wideband) which does not favor the phase measurement. Perhaps for this reason, only few studies of velocity profile measurement were made using this type of devices. One of such studies was carried out by Nguyen et al. [14]. A spike type pulser was used to measure the velocity profile using ACM. The damping control was adjusted to generate narrower band signals which favor the technique. Although the velocity was successfully measured, the system suffers with phase aliasing (also known as Nyquist limit) at higher velocities. Early researches used a priori flow knowledge to correct the phase aliasing [15,16]. However, those methods are not valid for velocities above twice the Nyquist limit and the prior knowledge is not always available. A well-known solution to this problem is the staggered pulse repetition frequency method that uses multiple pulse delays to correct the velocity [17,18]. With this method, the correct phase shift is estimated from measures obtained with two different pulse repetition frequencies. However, a specific delayed pulse control is required and that may be a constraint for NDT hardware pulsers.

Besides phase delay methods, there are also time delay techniques allowing the estimation of velocities beyond the Nyquist limits [19]. The cross-correlation method (CCM) is a well-known time shift technique that can overcome this limit as it relies on the time difference between pulses. In addition, wideband pulses of NDT pulsers improve the cross-correlation peak detection. The great disadvantage of CCM is the large amount of computing power needed to accurately compute the velocity [10] and for this reason it is not commonly used. Lai and Torp [20] developed an Extended Autocorrelation Method (EAM) that resolves the velocity ambiguity of ACM and with less computational cost than CCM by combining both techniques. The extended technique was only used to measure blood flow velocity using medical ultrasonic devices or simulated flow. As no specific hardware is required, EAM can be used with NDT pulsers.

Besides the spike pulser, there are also square wave type pulsers in NDT [21]. Those devices generate single cycle square waves that can be tuned to the resonant frequency of the transducer. This feature increases the SNR and improves depth penetration compared to spike pulsers. That is a desirable characteristic for ultrasonic velocity measurement as most of applications suffer with energy loss due to liquid-solid interfaces, particle scattering and depth attenuation. Thus, the use of square wave pulser for velocity profile measurement has a great potential in the field.

In this context, a study of EAM with NDT square wave pulsers is of great interest as it is a good alternative to measure velocities over the Nyquist limit. In this work, EAM technique is used with NDT square wave pulser to measure the velocity profile of a rigid-body motion of liquid inside a controlled rotating cylinder. First, the characteristics of a square wave pulser are presented. Next, the EAM is compared with ACM and CCM for velocities within and beyond the measurable range of ACM. The computational performance of each technique is also compared. Furthermore, the maximum value of measurable velocity of EAM and CCM are evaluated based on the number of times the velocity is beyond the Nyquist limit. Spatial and temporal parameters which have great influence on the measurement are also investigated. A recommended set of parameters are generated according to the maximum velocity desired.

## 2. Ultrasonic Velocity Profile Estimation Techniques

In this section, the basis of ultrasonic velocity profile is described. Additionally, all three velocity estimation algorithms used in this paper (ACM, CCM and EAM) are also presented.

### 2.1. Measurement Principle

**Figure 1:**
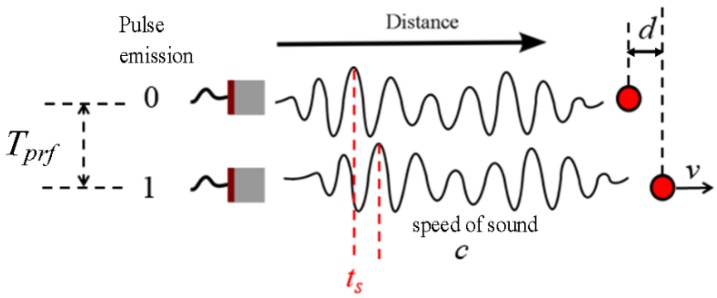
Schematic of the measurement principle. A moving reflector is captured by consecutive pulses and its displacement is measured.

Ultrasonic velocity profiler measures the particle delay between successive pulse emissions. Figure 1 shows a schematic of the measurement principle.

**Figure 2:**
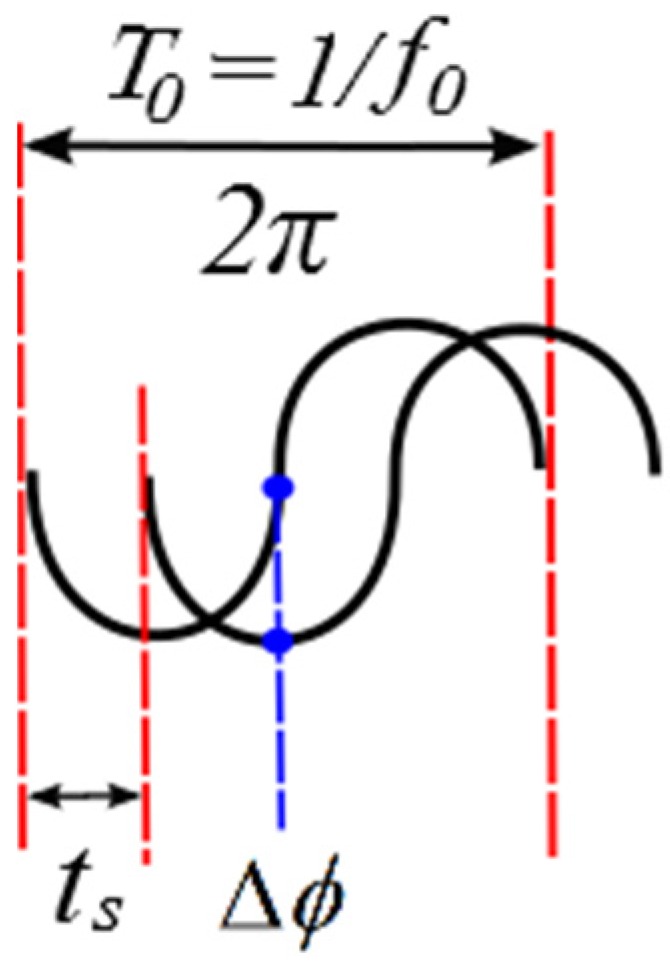
Phase shift or time shift used to measure the echo displacement.

From this figure, a moving reflector travels a distance *d* at a velocity *v* during the interval *T_prf_* between emissions, which results on the relation *v = d*/*T_prf_*. Due to reflector movement, the echoes will show a displacement represented by the time shift *t_s_* measured as the relation among the distance *d* and the sound speed *c*, given by *t_s_* = 2*d*/*c*. Combining both relations, the velocity is a function of the ultrasonic echoes displacements and it can be computed as:

(1) $$v=\frac{c\cdot f_{prf}}{2\cos\theta }t_{s}$$

where *f_prf_* = 1/*T_prf_* is the pulse repetition frequency. As ultrasonic waves are usually not travelling in the same direction of the particle movement, an angle *θ* is necessary to represent the right orientation. The echoes displacement can also be measured based on a phase shift (also called Doppler shift) Δ*φ* due to the ultrasonic wave nature. A simple relation between time shift and phase shift is shown in Figure 2.

Both time and phase shift are derived from the ultrasonic central frequency *f*_0_ as described in Equation (2):

(2) $$t_{s}=\frac{\Delta \phi }{2\pi \cdot f_{0}}$$

Replacing Equation (2) into Equation (1), we obtain the velocity estimation equation using the phase shift:

(3) $$v=\frac{c\cdot f_{prf}}{4\pi \cdot f_{0}\cos\theta }\Delta \varphi$$

Equation (3) is valid in the interval of ]–π,π]. Beyond this range, the phase shift is aliased as explained by Nyquist-Shannon theorem. Considering *f_prf_* in Equations (1) and (3) as a sampling rate, this limit corresponds to the Nyquist sampling limit. Therefore, the maximum measurable velocity for phase shift techniques is:

(4) $$v_{\max}=\frac{c\cdot f_{prf}}{4f_{0}\cos\theta }$$

Another well-established limit is the maximum measurable depth, *d_max_*, determined by the time-of-flight of the pulse to travel back and forth from the transducer:

(5) $$d_{\max}=\frac{c}{2\cdot f_{prf}}$$

Combining Equations (4) and (5), for *cosɵ = *1, velocity and depth are related according to:

(6) $$v_{\max}d_{\max}=\frac{c^{2}}{8\cdot f_{0}}$$

which represents the trade-off between velocity and depth due to the Nyquist limit.

### 2.2. Autocorrelation Method (ACM)

The most common velocity estimator is the autocorrelation method (ACM) which measures the phase shift (Equation (3)). The method was initially proposed by Namekawa et al. in the 80 s [22]. The phase relationship of a complex demodulated signal written as *r* = *x*(*n*) + *iy*(*n*) can be expressed by arctan(*y*/*x*), where *n* is the *n*th pulse emission. A phase shift may be approximated to the difference of discrete values from consecutive emissions of arctangent coordinates as shown in Equation (7):

(7) $$\frac{\Delta \phi }{\Delta n}=\arctan\left(\frac{y(n)x(n-1)-y(n-1)x(n)}{x(n)x(n-1)+y(n)y(n-1)}\right)$$

where the numerator and denominator of the arctangent function represent respectively the imaginary and real part of the autocorrelation function *R*() for *N* – 1 pairs of lines or emissions described as:

(8) $$R(1)=\frac{1}{N-1}\sum_{n=0}^{N-2}r^{*}(n)r(n+1)$$

where r* denotes a complex conjugate. The mean phase *φ_acm_* is estimated with:

(9) φacm=arctan(Im{R⌢(1)}Re{R⌢(1)})

where $\hat{R}\left(1\right)$ represents the averaged autocorrelation function. Im{} and Re{} are the imaginary and real parts.

Most commercial ultrasound equipment uses phase measurement since it has a very fast computational performance. Those systems enable the control of the number of cycles within an emission to generate narrowband pulses, which improve the phase measurement and the SNR. ACM main drawbacks are velocity ambiguity depending on Equation (6) and poorer axial resolution due to the longer pulse.

### 2.3. Cross-Correlation Method (CCM)

**Figure 3:**
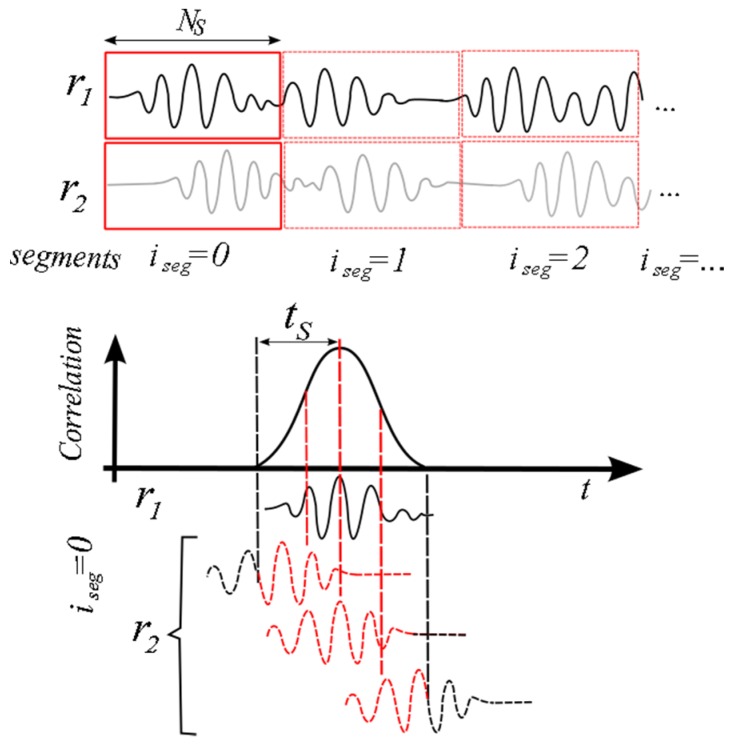
Segmentation of samples for the calculation of cross-correlation function.

The cross-correlation technique has been extensively used as a tool to investigate numerous digital signal processing applications involving time-delay estimation [23]. The technique was firstly proposed for ultrasonic velocity estimation by Bonnefous and Pesqué [24]. The measurement principle is illustrated in Figure 3.

Received echoes from subsequent pulses are divided into segment of *N_S_* samples. The ultrasonic signal pattern is correlated with the consecutive signal emission. The maximum value of the given correlation function is related to the time shift *t_s_*. The cross correlation among the received echoes of two emissions at each segment *i_seg_* is estimated by:

(10) $${\hat{R}}_{12}(n,i_{seg})=\frac{1}{N_{S}}\sum_{k=0}^{N_{S}-1}r_{1}(k+i_{seg}N_{S})r_{2}(k+i_{seg}N_{S}+n)$$

Because the signal is sampled at discrete times, the maximum of the correlation function may not coincide with the true position from the real signal. Therefore, the accuracy of CCM depends on a higher sampling frequency, with the penalty of increasing the number of calculations. Since the computational cost is already very high, it is preferred to do an interpolation scheme. The idea is to fit a curve which approximates the shape of the cross-correlation peak to obtain a fine estimate of its position. The method described in [10] fits a second-order polynomial to the three points at the peak. If the peak is found at lag *n_m_*, the interpolated peak is found at:

(11) $$n_{\mathrm{int}}=n_{m}-\frac{{\hat{R}}_{12}(n_{m}+1)-{\hat{R}}_{12}(n_{m}-1)}{2({\hat{R}}_{12}(n_{m}+1)-2{\hat{R}}_{12}(n_{m})+{\hat{R}}_{12}(n_{m}-1)}$$

where the interpolated estimate is given by:

(12) $${\hat{v}}_{\mathrm{int}}=\frac{c}{2}\frac{n_{\mathrm{int}}f_{prf}}{f_{s}}$$

A better resolution is obtained if the cross-correlation estimate is sufficiently noise free. A great advantage of CCM over ACM is that velocity estimation does not suffer with phase aliasing. The range depends on the search over the *N_S_* samples at the sampling frequency *f_S_*, which can be increased as needed. Equation (13) describes the largest detectable or measurable velocity *v_max_*:

(13) $$v_{\max}=\frac{c\cdot f_{prf}}{2\cdot f_{S}}N_{S}$$

A larger *N_S_* can enable the measurement of higher velocity, but it decreases the spatial resolution and increases the number of calculations. The computational cost is the main drawback of the method in comparison with ACM. As CCM measures time delays rather than phase changes, wideband pulses are desirable and can enable better spatial resolution to the velocity profile [25]. This characteristic is interesting in this study, since most NDT pulsers generate wideband pulses.

### 2.4. Extended Autocorrelation Method (EAM)

**Figure 4:**
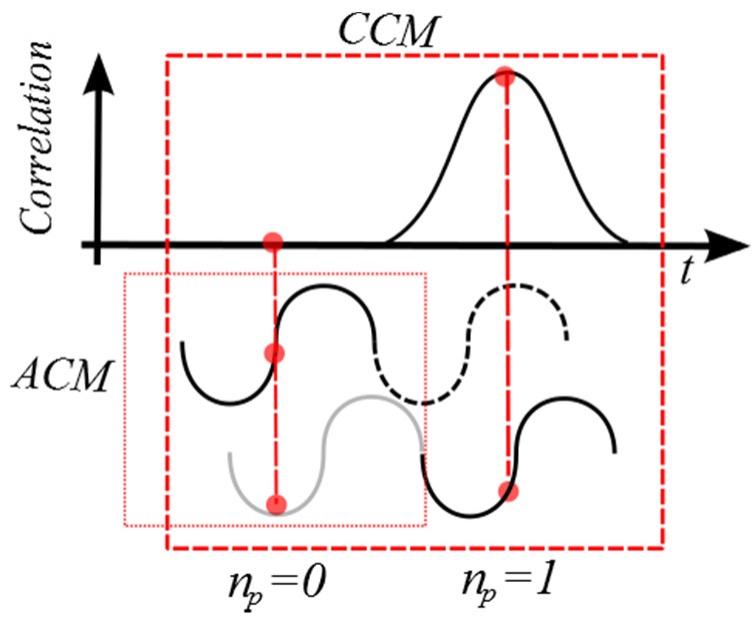
Schematic of EAM velocity estimator.

A combination of ACM and CCM is the main idea of the extended autocorrelation method EAM. Figure 4 describes a schematic of the principle.

An initial phase estimation is performed using ACM as described in Equation (3). Values beyond the interval ]–π,π] may be off by an integer number *n_e_* of 2π:

(14) $$\phi_{true}=\phi_{acm}+n_{e}2\pi$$

As CCM estimator can search over a larger range, a set of possible *n_e_* values […, −2, −1, 0, 1, 2, …] are used to find the true value. The search range is theoretically limited by the largest cross-correlation detectable velocity described on Equation (13). The EAM velocity is thus calculated with:

(15) $$v_{eam}=\frac{c\cdot f_{prf}}{4\pi \cdot f_{0}\cos\theta }\phi_{true}$$

Compared to CCM, the procedure greatly reduces the number of calculations as *n_e_* << number of time shifts. The other advantage is that the maximum measurable velocity is the same as CCM and it is not limited to the Nyquist sampling theorem as ACM. The trade-off between depth and velocity of Equation (6) is enhanced.

Although EAM uses both phase and time delay estimation to measure velocity, the technique behaves more like ‘CCM family’ due to the use of the magnitude of the correlation function to determine the correct delay candidate. According to Schlaikjer [26], a wideband pulse that favors CCM techniques is desirable to improve the EAM velocity estimation. In this context, NDT pulsers are a good match for the technique as they generally emit short pulses.

## 3. Square Wave Pulser

In the ultrasonic NDT field there are simple pulse-receivers that provide low cost ultrasonic measurement capability. An appropriate transducer and an acquisition system, allow those pulsers to provide the starting point for ultrasonic flaw detection, thickness gauging and materials characterization within the NDT field. The use of such pulsers may also be interesting for other ultrasonic applications such as fluid flow velocity measurement as proposed in this paper. In this context, there are pulse-receivers that employ spike excitation pulses and pulse-receivers that employ square wave pulses. Spike excitation pulses are optimized to applications involving testing of very thin materials. Square wave pulsers are useful in applications involving testing of thick or highly attenuating materials. This characteristic is desirable for velocity measurement in engineering applications, as most of the environments are highly attenuated due to liquid-solid interface (pipes), depth attenuation (open channel/larger pipes) or particle scattering (slurries/bubbles) [27].

**Figure 5:**
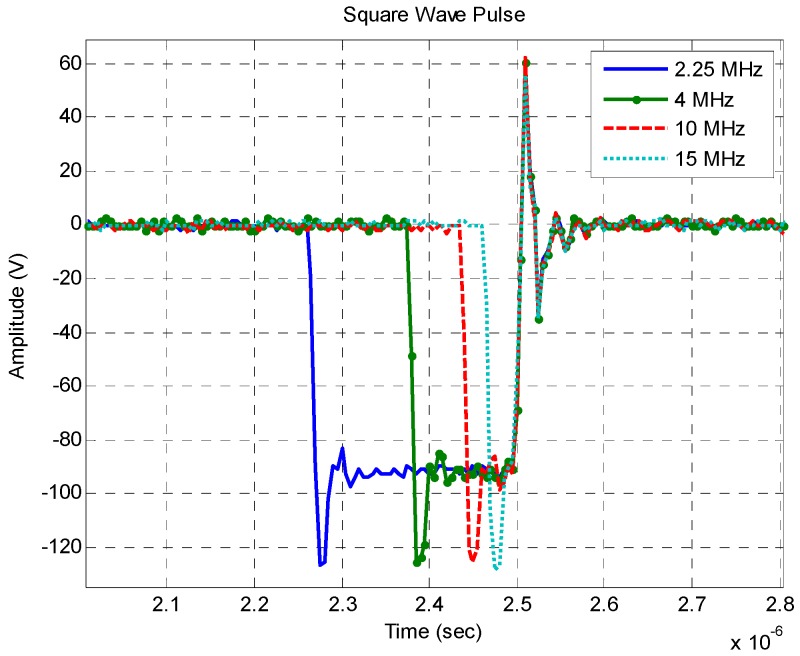
Square wave pulses tuned to half-frequency of transducers with 2.25, 4, 10 and 15 MHz center frequency.

**Figure 6:**
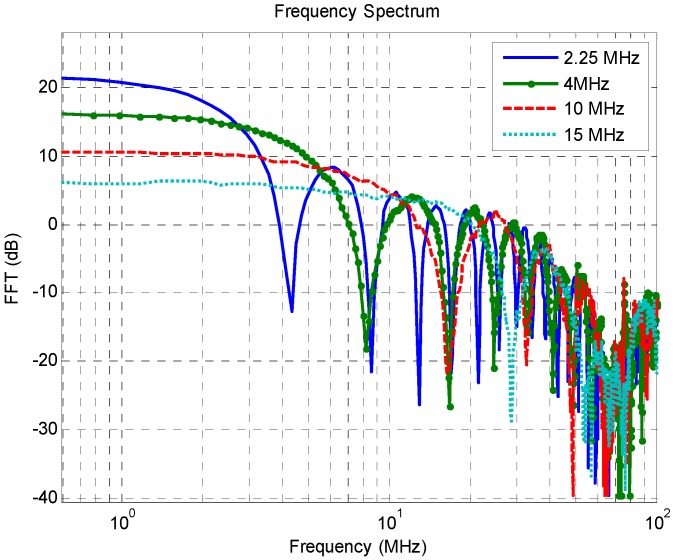
Frequency spectrum of square wave pulses tuned to half-frequency of transducers with 2.25, 4, 10 and 15 MHz center frequency.

The square wave pulser generates a signal characterized by a voltage fall followed by a voltage rise to the original state. The pulse voltage and pulse width can be controlled in this type of pulsers. The frequency spectrum is known as the sinc-function which, at low frequencies, shows a flat response and at higher frequencies presents a *1/f* decay with zeroes. By tuning the half period of the wave to that of the transducer resonant frequency, the energy is increased [28]. Figure 5 shows four square wave pulses obtained using a model 5077PR NDT pulser from Olympus (Tokyo, Japan) and a model MSO7000 wide band oscilloscope from Keysight (Santa Rosa, CA, USA). Those pulses were tuned to half frequency of 2.25, 4, 10 and 15 MHz. Figure 6 presents the same pulses in the frequency domain. As expected, the FFT amplitude is higher at the desired frequencies. In addition, comparing the 2.25 MHz tuned pulse with the 15 MHz pulse it is clear that increasing the frequency pulse leads to an energy decrease. According to the pulser manufacturer (Olympus), the square wave pulser is particularly advantageous when using transducers of 10 MHz or lower, and this can increase the signal gain by 12 dB or more if compared to spike pulsers using the same voltage settings. This characteristic is advantageous since most of UVP applications are within this frequency range.

**Figure 7:**
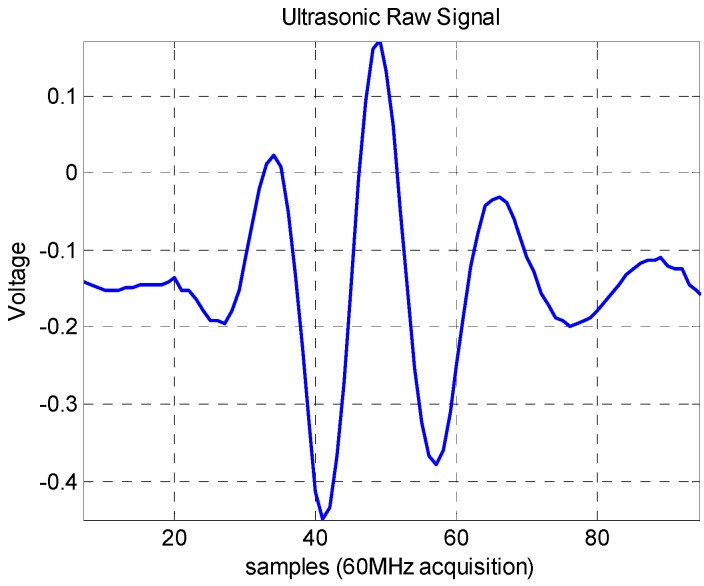
Ultrasonic raw signal of a wall echo.

ACM requires emissions with many cycles (narrowband) to measure phase difference. Recent studies using the technique to measure the velocity profile of fluid engineering applications, suggest four to eight cycles of the transducer basic frequency [29]. CCM needs well-defines peaks to correctly estimate the distance between consecutive pulses. As mentioned in the previous section, EAM desired pulse pattern should have both characteristics, but specially a well-defined peak to enable a better correlation between pulses. Figure 7 shows an ultrasonic echo from the NDT square wave pulser used in this work. A four cycle pulse with a well-defined peak is obtained. Such pattern satisfies the desired pulse conditions for velocity profile measurement for EAM and at the same time are not a constraint for ACM and CCM. More details regarding the NDT device are described in the next section.

## 4. Experimental Setup

In this section, the experimental apparatus used to evaluate the square wave pulser and the velocity estimation methods are presented. The comparison criteria and the different measurement conditions are also described.

### 4.1. Experimental Apparatus

**Figure 8:**
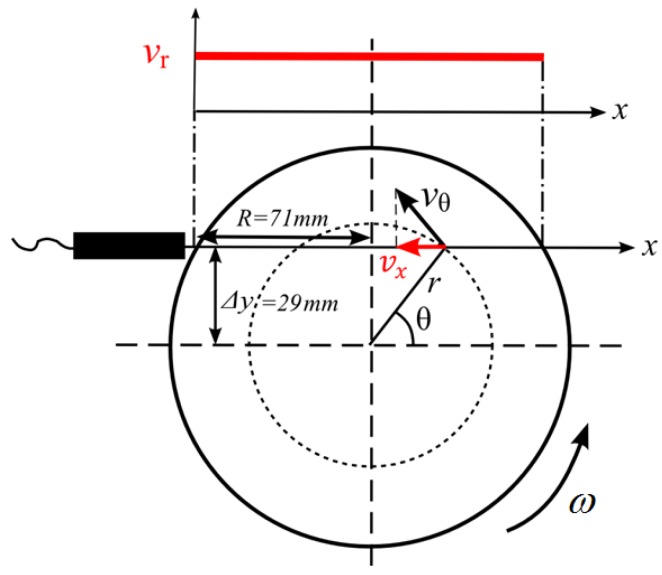
Velocity measured on the rotating cylinder experiment.

In order to evaluate the velocity estimation methods, it was used the rotating cylinder experiment. The rigid-body motion of the water inside the cylinder enables one-dimensional controlled velocities. It was one of the first experiments used to validate the ultrasonic velocity profile in the 1990s [1]. This configuration can be re easily realized in an experiment and is hydrodynamically stable. The velocity profile in this experiment is a flat line as described in Figure 8.

**Figure 9:**
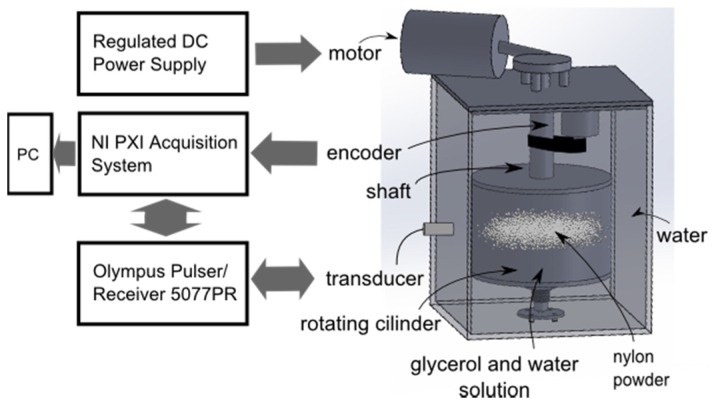
Block diagram of the velocity measurement system.

The velocity measured by the ultrasonic technique is in the transducer line direction, and it is represented by *v_x_*. Using trigonometric relations *v_x_* is related to *ω* by:

(16) $$v_{x}=\omega \Delta y$$

where *ω* is the angular velocity and Δ*y* is the distance between the transducer measurement line and the cylinder center, which for this work was 29 mm. The complete experimental apparatus is described in Figure 9. A rotating cylinder was filled with a solution of water/glycerol and tracer particles of 80 µm to 200 µm (Model 1A P82, EMS GRILTECH, Via Innovativa, Switzerland). Both liquid and particle have the same density (1.07 g/cm^3^). An electric motor was used to rotate the apparatus and the rotation speed was monitored with an encoder type sensor.

Ultrasonic pulses were generated and received using an Olympus model 5077PR Pulser/ Receiver and a 4 MHz transducer with 5 mm active diameter (Met-Flow, Lausanne, Switzerland). The pulse repetition frequency was set to 2000 pulses/second and the voltage is configured to −100 V. The main parameters of the equipment are presented in Table 1.

The signal was digitized using the acquisition system (model NI-5105 from National Instruments, Austin, TX, USA) with a 60 MHz sampling rate. A LabVIEW program controls the system and stores the data. A computer with Intel^®^Core™ i7-3770 3.4 GHz with 24 GB RAM with Matlab was used for signal processing.

### 4.2. Experimental Conditions and Methodology

The experimental conditions had the objective to validate the application of NDT square wave pulser for velocity estimation, and also the EAM capability of measuring velocities beyond Nyquist. The root-mean-square deviation (*RMSD*) is used as a metric to compare ACM, CCM, EAM and the cylinder reference velocity. The *RMSD* is normalized by the mean cylinder reference velocity in order to allow a fair comparison. This normalized *RMSD* is also called as Coefficient of Variation of *RMSD* (*CV_RMSD_*) and it is defined as:

(17) $$CV_{RMSD}=\frac{RMSD}{{\bar{v}}_{c}}\times 100\%$$

where ${\bar{v}}_{c}$ is the mean cylinder reference velocity and *RMSD* is given by:

(18) $$RMSD=\frac{1}{DT}\sqrt{\sum_{t=1}^{T}\sum_{d=1}^{D}{\left(v_{us}(d,t)-v_{c}(t)\right)}^{2}}$$

where *D* is the number of depths, *T* is the number of velocity estimates, $v_{us}(d,t)$ is the *t-*th ultrasonic velocity estimate at depth *d* and *v_c_*(*t*) is the *t-*th cylinder velocity estimate. A *CV_RMSD_* value of 0% indicates no difference between the estimated ultrasonic velocity and the cylinder reference velocity. The term *CV_RMSD_* will be used as a deviation metric in the Results section.

Velocities within and beyond the Nyquist limit (212 mm/s) were chosen to evaluate the estimators. Due to the limitations of the motor used, it was not possible to generate velocities faster than two times the Nyquist limit. An alternative to evaluate the maximum value of measurable velocity was to reduce the *f_prf_* according to Equation (4). The reduction in *f_prf_* decreases the maximum measurable velocity (Nyquist limit). For example, the maximum measurable velocity for *f_prf_* = 2 kHz is 212 mm/s, while using *f_pr f_* = 1 kHz the limit is reduced by half to 106 mm/s. Using this methodology, the original *f_prf_* = 2 kHz was divided by a decimation factor (2–7 times) enabling the validation of velocities up to six times the Nyquist limit.

Temporal and spatial resolution used for velocity estimation are also investigated. Some authors have studied those parameters for standard autocorrelation and cross-correlation methods. Loupas et al. [30] showed by extensive simulations that for autocorrelation and cross-correlation method, the number of samples (*N_S_*) should match the number of cycles of the basic frequency. The increase of the number of pulses (*N_P_*) also improved the measurement. Murakawa [31] investigated the *N_P_* influence on velocity estimation, and he also verified that higher values improved the measurement for engineering fluid flow applications. However, all analysis from the previous works were made for velocities under the Nyquist limit. In this context, a first test to evaluate EAM for velocities over the Nyquist limit is proposed. In this experiment, the *N_S_* and *N_P_* parameters are set to 128 samples, which follows the previous authors’ recommendations. A second study is carried out to investigate different temporal and spatial parameters combinations. In this experiment, the parameter *N_P_* took values from 2^4^ to 2^8^ pulses, while the parameter *N_S_* used values from 2^5^ to 2^8^ samples (according to Equation (13)). Those values were chosen as multiples of 2 to improve computational performance. Table 2 presents a summary of the parameters set evaluated in this work.

## 5. Results

In this section, the performance of the velocity estimation methods is discussed. First, the *CV_RMSD_* and the computational performance of ACM, CCM and EAM are compared. Velocities over one time the Nyquist limit are also evaluated. In the second experiment, the maximum measurable velocity is evaluated by increasing the Nyquist limit parameter. As temporal and spatial parameters have affected the results of the second experiment, a third experiment testing a variety of parameter values was performed.

### 5.1. Velocity Estimation and Computational Performance

**Figure 10:**
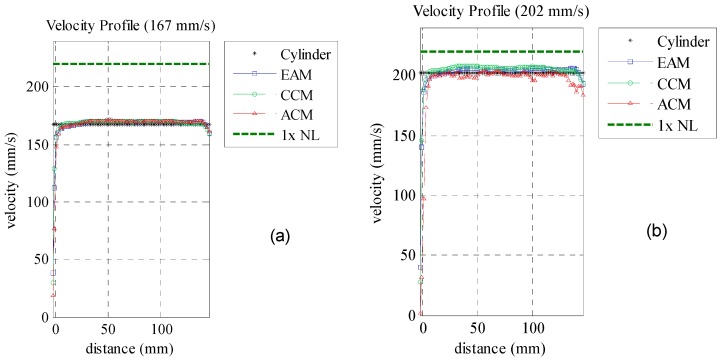

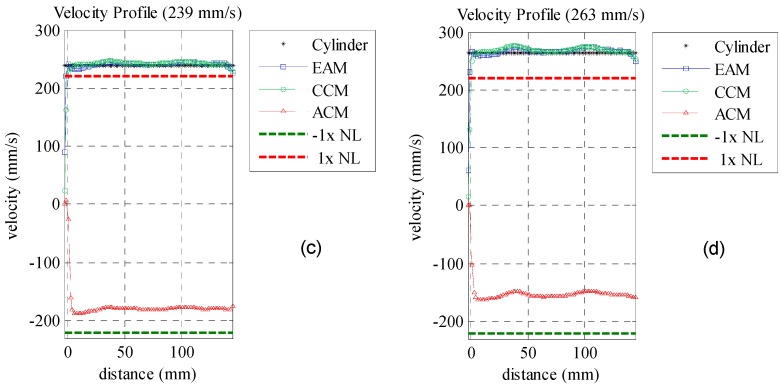
Velocity profile for a set of chosen cylinder velocities for ACM, CCM and EAM. (**a**) Cylinder velocity of 167 mm/s, under the Nyquist limit; (**b**) Cylinder velocity of 202 mm/s, under the Nyquist limit; (**c**) Cylinder velocity of 239 mm/s, over the Nyquist limit; (**d**) Cylinder velocity of 263 mm/s, over the Nyquist limit.

The mean velocity profiles over the distance are presented in Figure 10 and detailed in Table 3. ACM, EAM and CCM velocities were compared with the mean velocities obtained by the cylinder encoder (black dots). The *CV_RMSD_* is used to measure the difference between the methods. All velocity estimators presented deviations below 1% for velocities under the Nyquist limit. They are represented by the green circles, red triangles and blue squares in Figure 10. Velocities above this limit were not measured by ACM technique as phase aliasing occurred. On the other hand, CCM and EAM estimated the correct velocity with less than 2% deviation.

Table 4 shows the computational performance of all three techniques in seconds. Autocorrelation method has the best results and it is by far the fastest velocity estimator. The CCM estimator is the slowest technique due to the high number of operations required to calculate the cross-correlation. As EAM combines both ACM and CCM, the result presents an intermediate performance. It is 55 times slower than ACM, but 15 times faster than CCM.

Results show that square wave pulser is suitable for velocity profile measurement using phase (ACM/EAM) or time measurement techniques (CCM/EAM). The generated pulse shape successfully enabled velocity profile measurement with low deviation. In this scenario, EAM velocity estimator was validated to measure velocities beyond the Nyquist limit as well as CCM but with a better computational performance. This information is important since dealising techniques using multi-*PRF* or multi frequencies are not available in simpler NDT pulsers. Hence, EAM may be a good alternative to measure higher velocities with a reasonable computational processing time.

### 5.2. Maximum Measurable Velocity Based on Nyquist Limit

**Figure 11:**
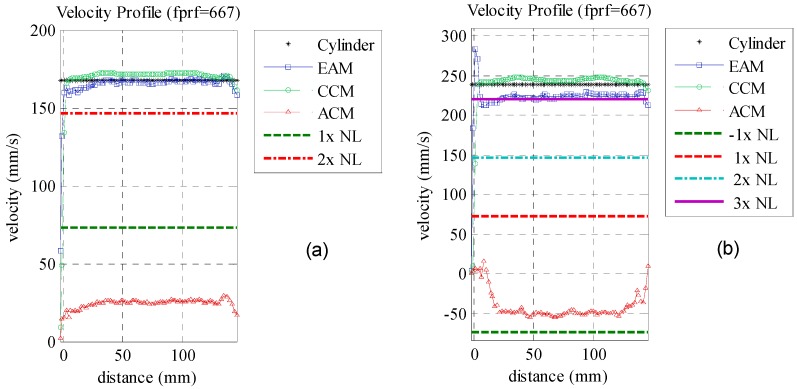
Velocity profile of three times decimated data. (**a**) Cylinder velocity of 167 mm/s is over two times *N_L_*; (**b**) Cylinder velocity of 239 mm/s is over three times *N_L_*.

Previously the EAM was compared with ACM and CCM for velocities beyond one time the Nyquist limit. Higher velocities were not validated due to the limitation on the used motor. An alternative to evaluate the maximum measurable velocity was to reduce the pulse repetition frequency by decimating the original signal. Figure 11 shows the velocity profile of three times decimated data for velocities of 167 and 239 mm/s (*f_prf_* = 667 Hz). The first velocity shown in Figure 11a, is over two times the *N_L_*. In this condition, autocorrelation aliased velocities are positive, differently from the negative values seen in Figure 10c,d due to the one time *N_L_* difference. EAM and CCM measured velocities with *CV_RMSD_* of less than 1% compared to the cylinder reference velocity. The velocity of 239 mm/s presented in Figure 11b is three times *N_L_*.

It is possible to verify that autocorrelation aliased velocities are negative again as there is one more *N_L_* difference. CCM and EAM presented 1.4 and 6.3% *CV_RMSD_*, respectively, a worse result compared to the two times *N_L_* scenario. A complete analysis of all measured velocities using *f_prf_* decimation factors with values ranging from two to seven times is shown at Table 5. The *CV_RMSD_* is used again as a performance index to compare the EAM and CCM velocity estimators. ACM is not evaluated as measuring *CV_RMSD_* of aliased velocities has no meaning. The over Nyquist limit parameter investigated in the previous experiment is also added to this table.

The results indicate that CCM velocities gave *CV_RMSD_* less than 2% up to over four times *N_L_*. After five times the *N_L_*, only the 263 mm/s was not measurable, and the other velocities have less than 2% *CV_RMSD_*. The results after six times *N_L_* presented deviations over 100% and they were considered non-measurable.

**Figure 12:**
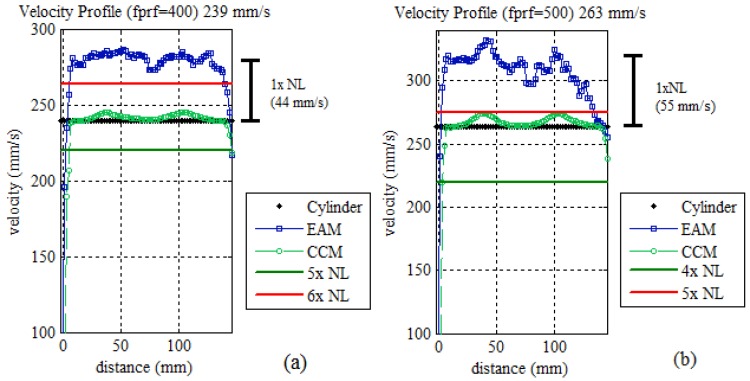
EAM and CCM estimated velocity profile for 239 and 263 mm/s cylinder velocity. (**a**) Velocity over five times *N_L_*; (**b**) Velocity over four times *N_L_*.

EAM obtained less than 2% *CV_RMSD_* up to over two times *N_L_*. Velocities over three times *N_L_* presented slightly worse *CV_RMSD_* values of around 5%–7%. Estimated values over four to six times *N_L_* presented more deviating results, with around 12%–17% of *CV_RMSD_*. Such an increase in the difference between the true value and measured value was investigated. The measured value presented an offset of one time the *N_L_* difference in both 239 and 263 mm/s velocities (Figure 12).

This is explained by a misdetection of the integer number n_e_ from Equation (14) by 1 time. If the n_e_ value is corrected, the CV_RMSD_ of both velocities would be less than 2%. Velocities beyond six times N_L_ were not measurable, presenting aliased velocities with deviations over 100%.

### 5.3. Temporal and Spatial Resolution Influence on Maximum Measurable Velocity

**Figure 13:**
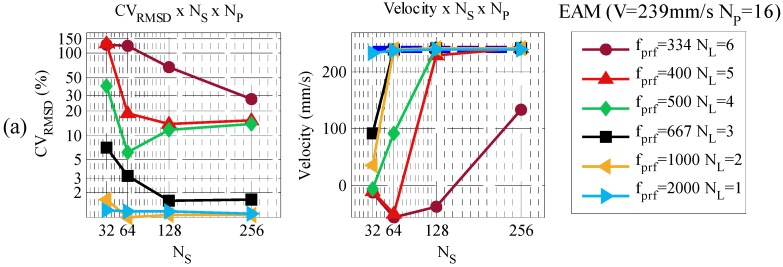

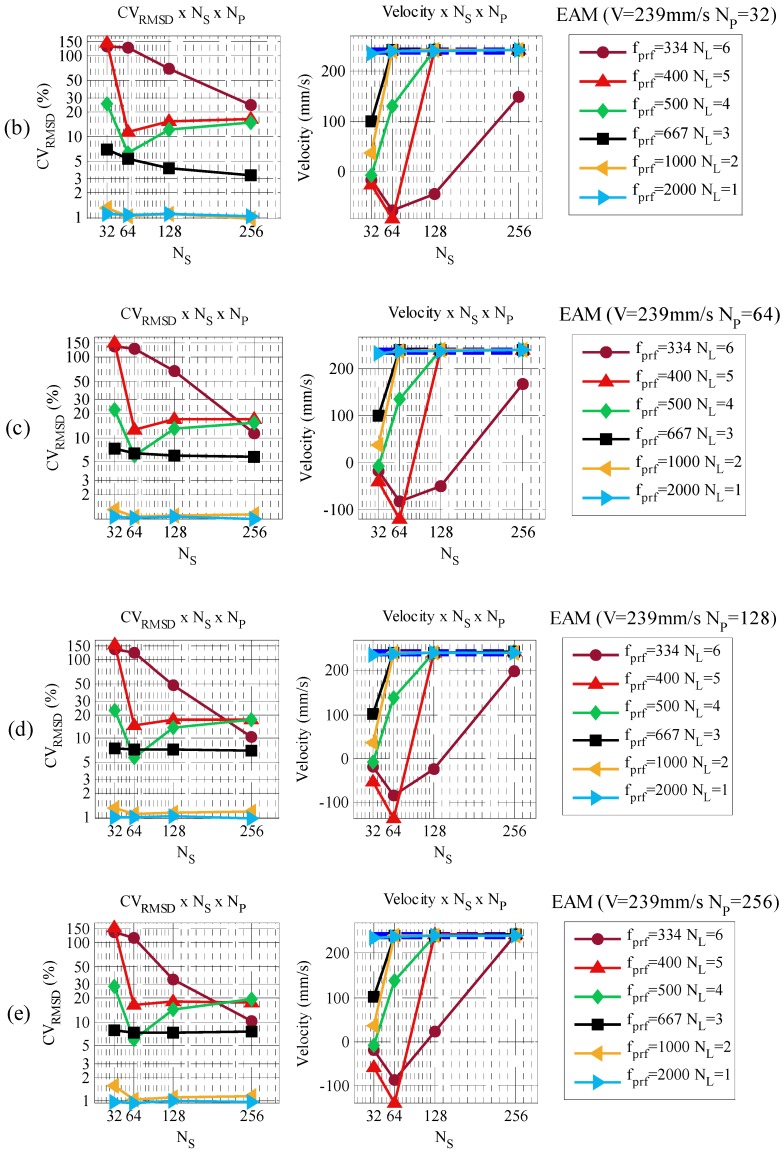
EAM Velocity profile and *CV_RMSD_* for a set of different *N_S_* and *N_P_*. (**a**) *N_P_* = 16; (**b**) *N_P_* = 32; (**c**) *N_P_* = 64; (**d**) *N_P_* = 128; (**e**) *N_P_* = 256.

**Figure 14:**
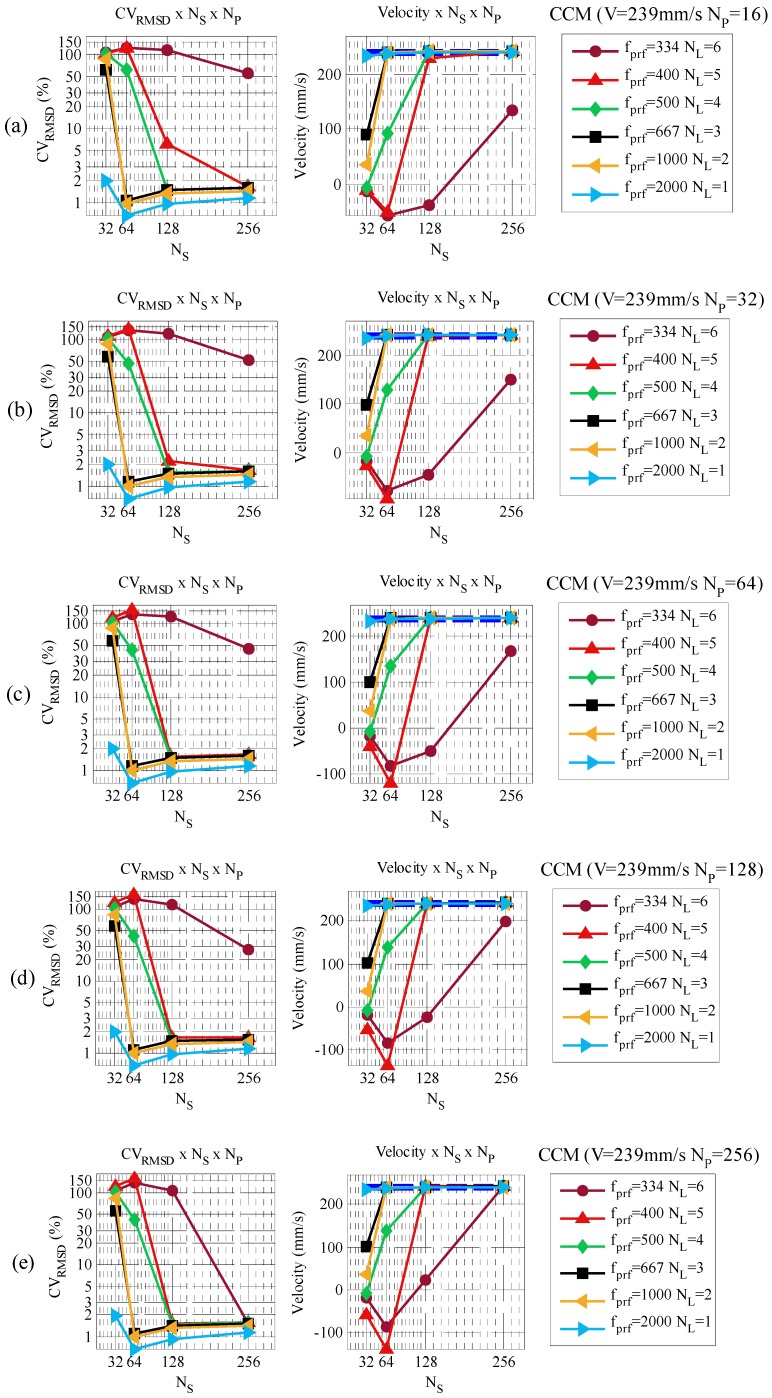
CCM Velocity profile and *CV_RMSD_* for a set of different *N_S_* and *N_P_*. (**a**) *N_P_* = 16; (**b**) *N_P_* = 32; (**c**) *N_P_* = 64; (**d**) *N_P_* = 128; (**e**) *N_P_* = 256.

The experimental results obtained in the previous section used 128 consecutive number of pulses *N_P_* (64 ms) and 128 number of samples *N_S_* (1.8 mm). During the experiments different results were observed as those parameters were changed. As there are no studies regarding those parameters for velocities beyond the Nyquist limit, another set of tests was proposed. In this section, a detailed investigation of *N_P_* and *N_S_* is carried out to verify the best parameters for EAM and CCM within different *N_L_* conditions. In this context, parameter *N_P_* took values from 2^4^ to 2^8^ pulses while parameter *N_S_* used values from 2^5^ to 2^8^. Pulse repetition frequency is also reduced to increase the over Nyquist limit *N_L_*. The same velocities from the previous experiment were tested, but as the purpose of the test was to evaluate *N_P_* and *N_S_* only one velocity will be shown. Figure 13 and Figure 14 present a test with different values of *N_P_*, *N_S_* and *N_L_*, using CCM and EAM. The cylinder reference velocity chosen was 239 mm/s. The other three velocities presented similar results.

CCM results show that higher *N_S_* and *N_P_* are best for all measured velocities. However, to improve the spatial and temporal resolution those parameters needed to be reduced. In these tests, a trade-off between spatial and temporal resolution was observed. Velocities of *N_L_* ≤ 4 are measured using *N_P_* = 16 and *N_S_* = 32, 64, 128 and 256 as *N_L_* increases. Increasing *N_P_* allows the reduction of *N_S_*. For example, velocities of *N_L_* = 3 are measurable using *N_P_* = 16 and a minimum *N_S_* of 64. The same velocity is measurable by increasing *N_P_* to 32 and reducing *N_S_* to 32. Velocities in the range of 5 ≤ *N_L_* < 7 required *N_P_* ≥ 128 and *N_S_* ≥ 256.

EAM presented two trend results. For *N_L_* ≤ 3 a lower *N_P_* and a higher *N_S_* are desirable. Measurements of less than 2% deviation are possible in this condition. Velocities in the range of 4 ≤ *N_L_* < 7 requires higher *N_P_* and a specific *N_S_* which is in the range between 32 and 256. Lower values of *N_S_* result on non-measurable velocities. Higher values of *N_S_* converge to a velocity aliased by one time the *N_L_* difference. This result indicates that phase aliasing are not completely corrected by EAM at higher velocities. A difference of 15–20% is observed in this situation, which at least is much better than no correction. The *N_S_* near 64 was the best condition in this experiment, but that may not be a general rule. In this context, 5 to 8% deviations are noted. Velocities of *N_L_* > 6 are not measurable regardless of the *N_S_* and *N_P_* employed. Based on this study a set of optimized spatial and temporal parameters is presented in Table 6.

## 6. Conclusions

In this work a NDT square wave pulser was used to evaluate an ultrasonic velocity profiler using an extended autocorrelation method. The technique combines both the autocorrelation and cross-correlation method, which were also evaluated in this work as a comparison parameter. All three velocity estimators were used to measure the unidimensional flow of a rotating cylinder. The root-mean-square deviation variation coefficient is used as a comparison metric between the reference velocity and the ultrasonic velocity estimators. Results show that NDT square wave pulser is suitable for velocity profile estimation. EAM, ACM and CCM obtained deviations lower than 2% for velocities under the Nyquist limit. Beyond one time this limit, CCM and EAM performed with the same deviation values. EAM has the advantage of being 15 times computationally more efficient than CCM and is a good alternative to overcome the maximum measurable velocity limit using NDT pulsers.

A more detailed analysis of the maximum measurable velocity of EAM and CCM was also investigated. The analysis was based on the number of Nyquist limit maximum measurable velocity and also on the temporal (*N_P_*) and spatial (*N_S_*) parameters for velocity estimation. CCM generally requires higher values of *N_P_* (up to 128 samples) and specially *N_S_* (up to 256 samples) for better results as velocity increases. The method could measure velocities up to six times *N_L_* with deviations close to 2%. EAM requires less *N_P_* and *N_S_* compared to CCM. Velocities up to three times *N_L_* were measured with deviations of less than 2%. Velocities beyond four times *N_L_* converged to velocities with one *N_L_* aliasing using higher values of *N_P_* and *N_S_*. The deviation observed in this situation were 15%–20%. Better results up to 5%–8% of deviation were obtained with lower specific values of *N_P_*. However that may not be a general rule. Finally, in this paper a NDT system was firstly used to measure velocities over the Nyquist limit using the EAM velocity estimator. The technique enables the measurement of real time velocities up to three times the Nyquist limit with a deviation lower than 2% and up to six times the Nyquist limit with a deviation of 15%–20%.

## 7. Future Work

The use of NDT pulsers to measure velocity profile could be extended to real pipes. Single phase and multiphase flow can be investigated using this system configuration. In this context, ACM, CCM and EAM could be compared. Other velocity estimators based on spectrum analysis and multi *PRF* could also be evaluated.

## Figures and Tables

**Table 1:** Olympus 5077PR main specifications.

Parameter	Value
Available Pulse Voltage (V):	−100, −200, −300, −400
Pulse Frequency Range (MHz):	15–20, 10, 7.5, 5–6, 3.5–4, 2–2.25, 1, 0.5, 0.25, 0.1
Pulse Repetition Rate (kHz):	0.1, 0.2, 0.5, 1, 2, 5

**Table 2:** Summary of experimental conditions.

Parameter	Value
Velocities:	167, 203, 239 and 263 (mm/s)
Temporal resolution (N_p_):	16, 32, 64, 128 and 256 (pulses)
Spatial resolution (N_s_):	32, 64, 128 and 256 (samples)
Velocities beyond Nyquist limit by signal decimation:	2, 3, 4, 5, 6 times Nyquist limit

**Table 3:** Summary of Velocity Results.

Mean Cylinder Velocity (mm/s)						
Encoder	ACM		EAM		CCM	
*v*	*v*	*CV_RMSD_* (%)	*v*	*CV_RMSD_* (%)	*v*	*CV_RMSD_* (%)
168	165	0.7	167	1.5	168	0.9
202	200	1.3	209	1.4	198	0.6
239	−185	---	239	1.5	243	0.8
263	−159	---	263	1.3	263	1.4

**Table 4:** Velocity Estimator Performance.

Processing Time (s)			
Velocity (mm/s)	ACM	EAM	CCM
167	0.34	18.6	166
202	0.32	17.9	168
239	0.33	18.2	165
263	0.33	18.1	166

**Table 5:** *CV_RMSD_* of CCM and EAM estimated velocities.

	EAM *CV_RMSD_* (%)				CCM *CV_RMSD_* (%)				
*f_prf_* (Hz)	Cylinder Velocity (mm/s)								<n>times *N_L_*
	167	202	239	263	167	202	239	263	
2000	1.4	1.3	1.5	1.3	0.5	0.9	0.9	1.8	0×
1000	1.1	1.7	1.2	1.4	0.9	1.1	1.2	2.2	1×
667	0.9	1.3	6.3	6.6	1.3	1.2	1.4	2.3	2×
500	5.8	6.0	12.8	16.1	1.3	1.3	1.4	2.3	3×
400	7.1	12.9	15.3	12.6	1.4	1.4	1.6	-	4×
333	13.0	15.0	-	-	1.4	1.3	-	-	5×
286	16.5	17.5	-	-	1.4	-	-	-	6×
250	11.1	-	-	-	-	-	-	-	7×

**Table 6:** Temporal and spatial parameters based on the velocity higher than <n> times the Nyquist limit.

Temporal and spatial parameters									
<n>times *N_L_*	*f_prf_* (Hz)	EAM				CCM			
		*N_P_*		*N_S_*		*N_P_*		*N_S_*	
		samples	ms	samples	mm	samples	ms	samples	mm
0×	2000	≥16	8	≥32	0.47	16/32	8/16	≥64/32	0.47/0.24
1×	1000	≥16	16	≥32	0.47	16/64	16/64	≥64/32	0.47/0.24
2×	667	≥16	42	≥32	0.47	16	42	≥64	0.94
3×	500	≥16	31	≥64	0.94	16/32	32/128	≥128/64	1.88/0.94
4×	400	≥64	160	≥64	0.94	16	40	≥128	1.88
5×	333	≥64	192	≥128	1.88	128	384	≥256	3.75
6×	286	≥64	224	≥256	3.75	128	448	≥256	3.75

Those *N_S_* and *N_P_* parameters are used to generate an improved EAM and CCM table. The *CV_RMSD_* results are shown in Table 7.

**Table 7:** *CV_RMSD_* of CCM and EAM estimated velocities based on optimized spatial and temporal parameters choice.

	EAM *CV_RMSD_* (%)				CCM *CV_RMSD_* (%)				
*f_prf_* (Hz)	Cylinder Velocity (mm/s)								<n>times *N_L_*
	167	202	239	263	168	202	239	263	
2000	1.5	1.4	1.5	2.1	0.7	1.4	1.1	2.0	0×
1000	1.0	1.5	1.2	1.5	1.0	1.2	1.3	2.2	1×
667	0.9	2.4	1.7	4.1	1.5	1.3	1.4	2.3	2×
500	1.9	7.7	5.8	7.0	1.4	1.4	1.5	2.3	3×
400	2.8	5.6	15.3	12.6	1.4	1.5	1.5	2.3	4×
333	5.8	15.0	10.4	-	1.5	1.4	1.5	-	5×
286	16.5	10.0	-	-	1.4	1.4	-	-	6×
250	11.2	-	-	-	1.4	-	-	-	7×
